# Patients’ Acceptance towards a Web-Based Personal Health Record System: An Empirical Study in Taiwan

**DOI:** 10.3390/ijerph10105191

**Published:** 2013-10-17

**Authors:** Chung-Feng Liu, Yung-Chieh Tsai, Fong-Lin Jang

**Affiliations:** 1 Department of Information Management, Chia Nan University of Pharmacy and Science, No. 60, Sec.1, Erh-Jen Rd., Jen-Te Dist., Tainan 717, Taiwan; E-Mail: fredliu@mail.chna.edu.tw; 2 Department of Obstetrics and Gynecology, Chi-Mei Medical Center, No. 901, Zhonghua Rd., Yongkang Dist., Tainan 710, Taiwan; E-Mail: yung0613@ms2.hinet.net; 3 Department of Medicine, Taipei Medical University, 250 Wu-Hsing Street, Taipei 110, Taiwan; 4 Department of Sports Management, Chia Nan University of Pharmacy and Science, No. 60, Sec.1, Erh-Jen Rd., Jen-Te Dist., Tainan 717, Taiwan; 5 Psychiatry Department, Chi-Mei Medical Center, No. 442, Sec. 2, Shulin St., South Dist., Tainan 70246, Taiwan

**Keywords:** patients, personal health records, technology acceptance model, physician-patient relationship

## Abstract

The health care sector has become increasingly interested in developing personal health record (PHR) systems as an Internet-based telehealthcare implementation to improve the quality and decrease the cost of care. However, the factors that influence patients’ intention to use PHR systems remain unclear. Based on physicians’ therapeutic expertise, we implemented a web-based infertile PHR system and proposed an extended Technology Acceptance Model (TAM) that integrates the physician-patient relationship (PPR) construct into TAM’s original perceived ease of use (PEOU) and perceived usefulness (PU) constructs to explore which factors will influence the behavioral intentions (BI) of infertile patients to use the PHR. From ninety participants from a medical center, 50 valid responses to a self-rating questionnaire were collected, yielding a response rate of 55.56%. The partial least squares (PLS) technique was used to assess the causal relationships that were hypothesized in the extended model. The results indicate that infertile patients expressed a moderately high intention to use the PHR system. The PPR and PU of patients had significant effects on their BI to use PHR, whereas the PEOU indirectly affected the patients’ BI through the PU. This investigation confirms that PPR can have a critical role in shaping patients’ perceptions of the use of healthcare information technologies. Hence, we suggest that hospitals should promote the potential usefulness of PHR and improve the quality of the physician-patient relationship to increase patients’ intention of using PHR.

## 1. Introduction

Healthcare policy has been changing. The World Health Organization (WHO) has advised health care institutions to replace passive therapies with active prevention measures in patient care. Such progress involves the active participation of patients in treatment programs and their contribution to electronic medical records (EMRs). C. Peter Waegemann, CEO of the Medical Records Institute (MRI), differentiated the five stages of electronic health care records (from the lowest to the highest level of sophistication) as automated medical records, computerized medical records, EMRs, electronic patient records, and electronic health records (EHRs) [1]. The fifth stage, EHR, emphasizes patients’ active participation in maintaining personal health records (PHRs) [1]. The concept of PHR systems (also known as PHRs) emphasizes empowering patients and increasing the interaction between physicians and patients to improve the quality of healthcare as a concrete method to implement EHRs, which can improve comprehensive data collection and clinical decision-making [2].

Telehealthcare, frequently used interchangeably with the terms telemedicine, telecare, and telehealth, is the use of medical information that is exchanged electronically from one site to another to improve a patient’s clinical health status. It provides the four fundamental benefits of improved access, cost efficiencies, improved quality, and patient demand [3]. Thus, the PHR system can be regarded as a kind of Internet-based telehealthcare implementation system and it is believed to have various potential benefits for patients, physicians, and the healthcare system overall [4]. However, patients may inadvertently input inaccurate data, or create inaccuracies by editing data obtained elsewhere [5]. Therefore, determining what functions should be provided and what format should be utilized for the data that are input by patients is essential to the successful implementation of a PHR system. Several studies have discussed personal cognition and acceptance of PHR from the perspective of the public or health consumers [6,7,8,9,10]. However, while exploring patients’ perceptions of PHR is an important issue of PHR research [11], such research is limited [12].

Infertile patients can be used as good indicators of the clinical utilization of PHR, as they are typically young, well educated, and familiar with the Internet [13,14]. The data from the prior studies cited above showed that approximately 60% of patients search for medical information about infertility on the Internet. Another advantage of PHR in infertility treatment is that detailed and private records about basal body temperature and sexual life can be recorded and reviewed through a PHR system. Patients can conveniently provide their daily records using a PHR system instead of periodically and publicly handing in their records to physicians or nursing staff in a hospital. Accordingly, the use of PHR in infertility treatment has practical advantages.

Nevertheless, owing to the need for complicated information communication technologies (ICTs) and particular physician-patient behaviors, the adoption of PHR requires a high-cost investment, and knowing the factors that affect patients’ intentions to use PHR is crucial to hospitals’ being able to optimize development strategies. To identify these factors, a web-based PHR system that is suitable for use by infertile patients was developed in this work with the cooperation of healthcare information management scholars and experts in psychiatry and infertility. Then, a self-reported survey of infertile patients in a large hospital was performed. Since the Technology Acceptance Model (TAM) [15] is one of the most used models for determining an individual’s acceptance of technology and a variety of studies in healthcare have investigated the public’s or patients’ acceptance of healthcare information systems based on TAM (or extended TAM), this study also used TAM, incorporating an innovative construct “physician-patient relationship (PPR)” as a theoretical basis. The possibility of caring for patients outside of hospitals with the support of information technology has not been studied extensively, and studies of patients’ attitudes toward the use of technology are especially lacking. We hope that the results of this study can contribute to academic and practical knowledge in healthcare information management and medical informatics.

## 2. Literature Review and Hypotheses Development

### 2.1. Personal Health Records

#### 2.1.1. Definitions and Functions of PHR

Numerous definitions for PHR have been provided by several professional organizations [5,16,17]. The Healthcare Information and Management Systems Society (HIMSS) provided a comprehensive assessment, in which it defined ePHR (which also can be shortened to PHR) [17] as follows:

“An electronic Personal Health Record (“ePHR”) is a universally accessible, layperson comprehensible, lifelong tool for managing relevant health information, promoting health maintenance and assisting with chronic disease management via an interactive, common data set of electronic health information and e-health tools.”

Kim and Johnson [18] developed helpful criteria for evaluating a PHR system in terms of the following five functions: (1) providing web-based access to personal medical information; (2) providing an organized summary of personal medical information for presentation to healthcare providers; (3) serving as a portal to patient-specific consumer-level healthcare information; (4) providing interpretive information about the results of laboratory tests and diagnostic studies; and (5) serving as a database of information for patient-specific self-monitoring and disease management.

To fill this potentially significant niche, several commercial PHR platforms have been developed. These include WebMD’s MyHealthRecord, WellMed’s Personal Health Manager, and CBSHealthWatch’s About My Health. Microsoft and Google also both announced online PHR products in 2007 and 2008, respectively (MS HealthVault and Google Health) to help the public conduct general health self-management (however, Google announced the withdrawal of Google Health in 2011).

#### 2.1.2. PHR for Infertility

Infertility is defined as a married couple being unable to conceive a baby after at least 12 months of regular sexual activity without contraception. In Taiwan, Department of Health reported where the birth rate has declined gradually, approximately 15% of married couples experience infertility. Infertility treatment is a long-term process with no guarantee of success. Several routine data of infertile patients, such as their living status (whom they live with), frequency of sexual intercourse, and variations in basal body temperature, are essential information that physicians can refer to when treating infertility. However, the up-to-date data cannot be obtained completely during outpatient visits. Besides, many studies have indicated that before or during infertility treatment, patients experience high mental stress, negative thoughts, and low self-esteem, as well as emotional disturbances, such as anxiety and depression [19,20,21,22]; thus relaxation training for patients can be helpful during the infertility treatment as well. Again, this relaxation training cannot be obtained completely during infertility outpatient visits. Therefore, a PHR system enables infertile patients to record their routine health data as well as to practice self-relaxation and record their corresponding perceptions during each practice.

### 2.2. Technology Acceptance on PHR Adoption

Most studies of PHRs have investigated their functional characteristics and technological schemes or structures for their implementation [23,24,25,26], or surveyed statistical reports of outcomes of their use, determining such factors as frequency [9,27] and satisfaction [28]. Some studies have discussed personal cognition and acceptance by the general public [6,7,8,9]. Despite of the fact that a conceptual framework has been proposed to explain the adoption of PHR by older adults with chronic illness [29], empirical analyses of the acceptance of patients of PHRs and their perceptions thereof are crucial to PHR research [11] but few have been conducted [12].

Shaping users’ or potential users’ perceptions of technology adoption has been a major research issue in previous decades. The TAM, which was proposed by Davis and colleagues [15,30], was the primary model used in these studies. Although acceptance of technology is a thriving field in management information systems (MIS) research, it has been considered less in the healthcare sector than in other information-intensive sectors [31]. Numerous studies that involve healthcare professionals have evaluated the relationships within TAM or extended TAM [32,33,34]. Other studies, targeting patients and the public as users, have studied perceptions of healthcare information systems based on TAM [35,36,37]. An empirical investigation of general consumers’ adoption of USB-based PHR also found that TAM provides an appropriate theoretical basis [10]. Accordingly, TAM is utilized as the baseline model in this study and the original hypothesized relationships of TAM in the PHR context follow:

Hypothesis 1: A positive relationship exists between PEOU and PU in the PHR context.
Hypothesis 2: A positive relationship exists between PEOU and BI in the PHR context.
Hypothesis 3: A positive relationship exists between PU and BI in the PHR context.

### 2.3. Physician-Patient Relationship and Technology Acceptance

Physician-patient communication serves as a modality for educating patients on their health care, including disease evaluation, diagnosis, and prognosis. Components of effective physician-patient communication during a medical visit include setting the right tone, interpreting communication cues accurately, and active listening [38]. Effective communication is essential to delivering quality patient care and establishing physician-patient relationships (PPR) [38]. Much of the literature on PPR has defined the term, so PPR is operationally defined herein as “the extent of familiarity, trust, and interaction between physicians and patients in the context of healthcare planning.” Undoubtedly, PPR has always been considered to be a factor that significantly affects the quality of medical care [39,40,41].

A focus group study found that patient-driven communication and mutual trust between physicians and patients are two important determinants of patients’ perceived usefulness of online EMRs [42]. Ventres and colleagues posited that the relational effect, which refers to the perceptions of EHRs by physicians and patients and the effect of such perceptions on their use, is a key factor that can shape the development and maintenance of PPRs, as well as influence their implementation [43]. Several studies have also indicated that PPR is related to intentions to use health-related information technology [44,45]. Based on the preceding discussion, we suspect that the PPR has a direct effect on patients’ acceptance of healthcare technology. Hence, we propose the following hypothesis:

Hypothesis 4: A positive relationship exists between PPR and BI in the PHR context.

## 3. Method

### 3.1. The Web-Based Infertile PHR System

A web-based PHR system for patients with infertility was implemented for therapeutic purposes. The requirements of this system were determined by a research team (a psychiatrist, an infertility physician and a healthcare information management researcher with a PhD. The system was implemented on a PC server using Microsoft Visual Studio with ASP.NET and the Microsoft SQL Server database system, whose homepage displayed three main frames for visitors (patients):

(1) Main menu: (1a) The upper right-hand corner of the website provides therapists’ suggestions based on the outcome of the patient’s last self-relaxation training; (1b) the middle of the right-hand side of the website presents items that should be personally recorded and evaluated during the current session; and (1c) the bottom right-hand corner presents the items that were not evaluated by the patient in the previous week and reminds users to complete the evaluation.
(2) Record history searching function: The search function, located in the upper left-hand corner of the web site enables patients to look up historical records and explanations and suggestions that have been previously provided by the relaxation training therapist.
(3) Message board function: The bottom left-hand corner of the website provides a platform for patients and care teams (physicians and therapists) to ask questions, leave messages, and post notifications. The research team’s e-mail address and phone number are posted on the PHR Web site. Patients who participate in the program can contact the research team at any time via e-mail or telephone.

**Figure 1:**
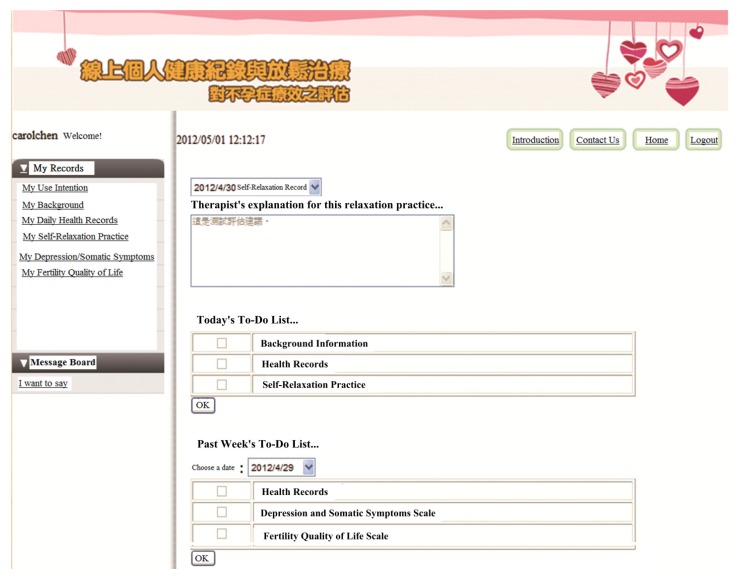
The post-login page of the PHR system.

The system enables the recording of a daily PHR, including patients’ living status (with whom they live), basal body temperature, and whether they engage in sexual intercourse with their spouse. This system also provides instructional audio material online to help with progressive muscle relaxation [46] around 20 min long: patients can listen to it while simultaneously practicing relaxation. After completing their daily relaxation, patients should complete the self-evaluation questionnaire regarding each practice. The system also provides other self-evaluation tools (Depression and Somatic Symptoms Scale (DSSS) [47] and the Fertility Quality of Life Scale (FertiQol) [48]) for patients, which should use them once per one week and once every two weeks, respectively. The physicians and therapists can check these patients’ self-evaluations online to support continual treatment. Figure 1 presents the homepage of the system as seen after patients have logged in.

For reasons of privacy and confidentiality, rather than recording information that can identify a participant (such as his or her real name), the PHR system stores only the patient’s user account, which was used to link uniquely the patient’s personal health records. Information that links accounts with real names is recorded in a written document that is kept by the infertility physician who was caring for the patients (participants) in this study. Although other researchers can access the health records associated with all accounts, however, they cannot identify the records of individual patients. Additionally, the secure sockets layer (SSL) network security encryption mechanism was adopted to ensure that patients’ health records were completely protected whenever they were transmitted over the Internet.

### 3.2. Research Model

**Figure 2:**
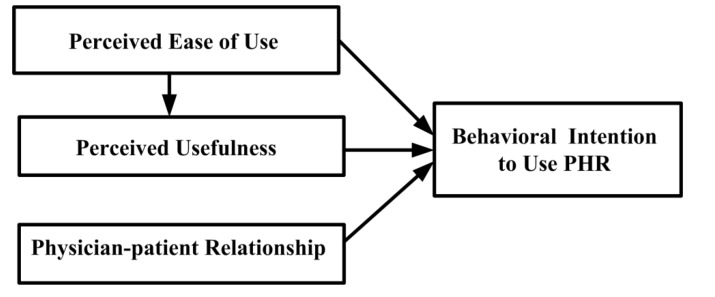
Research model.

Numerous researchers have incorporated other constructs according to the study context to extend TAM and better predict people’s acceptance of technology. Based on the above literature review, the PPR concept is incorporated into the TAM herein as an extending variable to help to evaluate patients’ perceptions of, and intentions to use, PHR. Figure 2 shows the research model.

### 3.3. Setting and Ethics Approval

The participants of this investigation were infertile patients who were recruited from a 1,300-bed medical center in Taiwan. Owing to the importance of protecting the participants’ rights and privacy, a formal ethics-based approval of this study was obtained from the Institutional Review Board (IRB) of the study site. The participants should satisfy the following five criteria:

(1) Aged between 25 and 45 years.
(2) Diagnosed with infertility by a physician.
(3) Have not adopting nor were adopting children.
(4) Have not received cognitive behavioral therapy of any form within the two months before the study.
(5) Could access the Internet at home.

### 3.4. Instrument Development

In this study, each patient’s acceptance of the PHR system was measured with reference to their PEOU, PU, and BI using three, five, and two questions, respectively. These questions were adapted from the TAM literature [15,30]. All items were measured using a 5-point Likert scale (where 5 denoted strong agreement and 1 denoted strong disagreement). The three PEOU items were, “I think that the PHR system is flexible to use”, “Learning to operate the PHR system is easy for me”, and “I can easily get the PHR system to do what I want it to do”. The five PU items were, “Using the PHR system improves the effectiveness of my health care”, “Using the PHR system decreases the complexity of my health care”, “Using the PHR system increase the quality of my health care”, “Using the PHR system for my healthcare is a good idea”, and “I find the PHR system useful in my healthcare”. Five items that are taken from Q21, concerning a patient’s “Last GP Appointment” in the GP Patient Survey [49] that is used periodically by the Department of Health, U.K., to elicit patients’ experiences, were utilized in this study to evaluate the PPR concept. Patients were instructed to respond to the question items using a 5-point Likert scale (where 5 denoted very good and 1 denoted very poor); the five items, concerning the actions of the physician, were (1) giving you (the patient) enough time; (2) listening to you; (3) explaining tests and treatments; (4) involving you in decisions about your care; (5) treating you with care and concern.

The draft of the questionnaire that was used in this study comprised a cover page and several question items. The cover page briefly introduced the purpose of the study and practically defined the PHR. This draft was refined by experts, comprising two health care information management researchers, a psychiatrist, and an infertility physician. Then, a pretest was conducted with five adult women, whose opinions were compiled into a reference for modification to obtain the final version of the questionnaire.

### 3.5. Procedures

An infertility physician (a researcher in this study) introduced the investigation and invited them to participate in the study during outpatient visits. The physician directed willing patients to visit a nearby secured office room, where three well-trained research assistants were standing by to explain the study in detail, including the need for participants to record some private or sensitive information, such as about their sexual lives, in the PHR system. Patients who understood and were willing to participate were required to complete two consent forms—one for the hospital and one for themselves. Then, research assistants instructed the patients to create an account and password and immediately online via an Internet-connected notebook computer. The research assistants used this account to login and explained all of the system functions and operating procedures to ensure that the patients completely understood how to use the system. The assistants were also obligated to inform patients that they could withdraw from the study at will and their following care plans would not be affected if they did so. Participants would lose the ability to use the system if they failed to make or update any health record (concerning basal body temperature/sexual life and self-relaxation practice) for more than seven consecutive days. After the participants had used the system for a month, they were reminded to complete the questionnaire in the PHR system regarding their acceptance of it.

## 4. Data Analysis and Results

### 4.1. Descriptive Statistics

Ninety patients were recruited as participants in this study. A total of three patients withdrew from the study, 35 patients lost their access permissions before the end of study because they did not use the system for seven consecutive days, and 52 people completed the study. Finally, 50 questionnaires were validly completed, yielding a response rate of 55.56%. Of the 50 patients whose responses were valid, most had a university degree (n = 25, 50.00%), 20.00% (n = 10) had a college degree, 18.00% (n = 9) had a high school degree, and 12.00% (n = 6) had a master’s degree. Most of them were aged between 31 and 35 years (n = 27, 54.00%), 24.00% were between 36 and 40 years (n = 12), and 22.00% were between 26 and 30 years (n = 11). 

We also collected and collated 38 feedbacks for why they failed to complete the study: (1) subjects were too busy or forgot to use the system regularly (n = 30, 78.9%); (2) subjects didn’t think that the PHR was helpful for their therapy (n = 6, 15.8%); and (3) subjects felt that the PHR was not stable to use (n = 2, 5.3%).

As indicated in Table 1, the participants’ perceptions of PEOU and PPR were positive (with mean values of 4.19 and 4.07, respectively), and their perceptions of PU were moderately high (with a mean value of 3.71). Their overall intention to use PHR was positive (with a mean value of 3.89).

**Table 1:** Descriptive statistics of criteria for determining quality of responses.

Construct	Item	Loading	Mean	Std. Dev.	Cronbach’s α	CR	AVE	Communality	Redundancy
Perceived Ease of Use (PEOU)	PEOU1	0.72	4.21	0.64	0.84	0.91	0.77	0.77	
	PEOU2	0.93							
	PEOU3	0.95							
Perceived Usefulness (PU)	PU1	0.90	3.72	0.55	0.92	0.94	0.76	0.76	0.12
	PU2	0.86							
	PU3	0.88							
	PU4	0.81							
	PU5	0.91							
Physician-patient Relationship (PPR)	PPR1	0.79	4.06	0.74	0.94	0.95	0.80	0.80	
	PPR2	0.94							
	PPR3	0.93							
	PPR4	0.91							
	PPR5	0.90							
Behavioral Intention (BI)	BI1	0.98	3.89	0.66	0.94	0.97	0.95	0.95	0.16
	BI2	0.97							

### 4.2. Reliability, Validity and Model Fit

The partial least squares (PLS) technique was used in this study to evaluate the measurement and structural models [50]. Principal component analysis of the PLS was performed to ensure the unidimensionality of the three constructs PU, PEOU, and PPR. The factor loadings of all of the items exceeded 0.72 (which exceeded the cut-off of 0.7, which was suggested by Fornell and Larcker [51]) and were significantly associated with only one latent variable, indicating conformance to unidimensionality [52].

Cronbach’s α of all the constructs exceeded the suggested cut-off value of 0.7, and the composite reliability (CR) of all of them exceeded the suggested cut-off value of 0.6; these results all indicated that the measurement satisfied the reliability criteria [53]. Fornell and Larcker [51] suggested using the average variance extracted (AVE) as a measure of convergent validity. Table 2 demonstrates that the AVEs ranged between 0.76 and 0.95, exceeding the cut-off value of 0.5 [51], suggesting satisfactory convergent validity. Additionally, none of the construct intercorrelations exceeded the square root of the AVE of the constructs, establishing discriminant validity [51]. Overall, all of the constructs in this study exhibited sufficient convergent and discriminant validity.

**Table 2:** Correlation matrix of constructs.

	PEOU	PPR	PU	BI
Perceived Ease of Use (PEOU)	0.88			
Physician-patient Relationship (PPR)	0.36	0.89		
Perceived Usefulness (PU)	0.39	0.43	0.87	
Behavioral Intention (BI)	0.50	0.56	0.67	0.98

Note: Bold numbers on diagonal are square roots of AVEs of constructs.

Goodness-of-Fit (GoF) was employed to judge the overall fit of the PLS model, which is computed as the geometric mean of the average communality and the average R square. GoF is normed between 0 and 1, where a higher value represents better path model estimations. For our model the GoF value was 0.55, which exceeds the cut-off value in comparison of baseline value as GoFsmall = 0.1, GoFmedium = 0.25, GoFlarge = 0.36 [54] indicating that our model had substantial predictive power.

### 4.3. Hypotheses Testing

**Figure 3:**
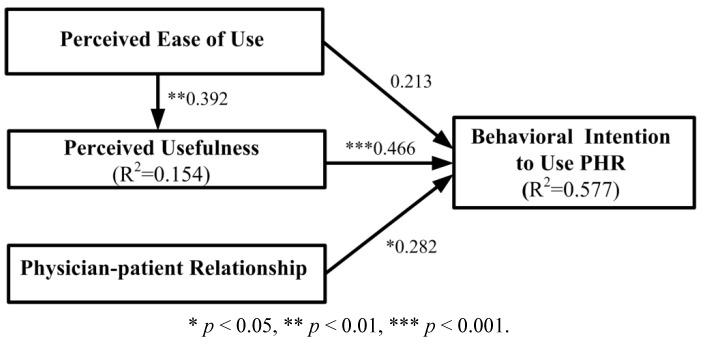
Results of the path modeling analysis.

The statistical significance of the parameters in the structural model was tested using the bootstrapping resampling procedure of the PLS method. With a significance of 0.05 or better, the results revealed that the patients’ perceptions of PU and PPR were positively associated with their behavioral intentions to use PHR, with an explanatory power of 57.7%. Furthermore, PEOU explained approximately 15.4% of the variance in PU. These results support Hypotheses 1, 3, and 4. Figure 3 presents the standardized path coefficients of the causal path.

* *p* < 0.05, ** *p* < 0.01, *** *p* < 0.001.

## 5. Discussion and Suggestions

### 5.1. Behavioral Intention

The results of this study indicate that patients generally exhibited a moderately strong intention to use the PHR system (mean = 3.89). This finding is similar to those of previous investigations of patients’ acceptance of assistive medical technologies [35,55,56]. A report from the USA found that, despite the currently low adoption of PHRs, patients are interested in PHR applications [57]. This finding implies that patients tend to accept the use of electronic clinically external communication channels to interact with their healthcare professionals, and supports the development of patient-involving information systems to supplement medical visits. Patient-side applications should be promoted by researchers and healthcare stakeholders to improve patient care.

### 5.2. Effect of PEOU on PU

We found that patients’ PEOU is directly related to their PU. This finding conforms to the TAM’s theoretical basis. Consistent with previous patient technology acceptance studies [36,58], the results of this study suggest that patients are more likely to perceive the PHR as useful if they feel that the PHR is easy to use. The PU is measured regarding the improvement to health care procedures and quality by using the functions in an information system. Evidently, patients may not experience the complete functions of a system without an ease-of-use interface.

The PHR system that is developed in this study is web-based design and is operated in a manner similar to general web-based network services (which use, for example, hyperlinks and drilldown). One of the inclusion criteria of this study was that the participant must be able to use Internet services. Thus, the interface operation was expected to be free of obstacles for them. Furthermore, responses to all questions for self-completion required responses on a 5-point scale, which users could just click, and users could leave comments by freely typing. Users could easily use all of the functions and understand their purposes. Hence, PEOU was predicted to have a significant effect on PU. 

Since patients’ technological literacy varies, we suggest that the ease-of-use and uniformity of the operational processes of the PHR, and its consistency with typical web-based services, should be emphasized

### 5.3. Effect of PEOU on BI

Numerous studies of the general public or patients’ acceptance of health care technology services have shown that PEOU has a direct effect on BI. However, this study could not confirm this finding [36,58,59]. A similar survey, Jian’s study [10] relating to consumers adoption of USB-based PHR, also did not verify this causal relationship. 

The convenience of the user interface is a factor that critically influences decisions to use organizational application systems and electronic business systems or services that exist wide providers or alternatives [60,61,62,63]. If the operating process or interface design of a system is unfriendly, then users may criticize its providers or developers and may even refuse to use the system and seek alternative products or services. However, ease of use may not be directly related to intention to use systems with specific purposes that have few replacements or alternatives, such as online banking services [64,65] and the spoken dialogue systems that are used by clinicians [66]. A medical support system for use by patients is a system with specific purposes. Patients use such systems as required by their health needs either spontaneously or following the advice of medical professionals. Patients most concerned about whether the content and services provided by the system are useful (for improving their health or alleviating their pain and suffering). Therefore, problems with the system’s interface are not a major consideration.

In this study, patients hoped to improve their health (improved infertility and reducing stress) by through using the functions of the system. Accordingly, the ease of use of the system was not a great concern for the participants. During the study, the participants rarely reported problems related to the user interface, suggesting that placing excessive emphasis on the appearance, aesthetics or operational convenience of the interface is unnecessary in the design of a PHR system; instead, the design should focus most on the factors that the patients most value, such as the relevance of the system’s functions.

In this study, patients hoped to improve their health status (enhancing infertility treatments and reducing stress) through using the functions provided by the system. Therefore, the ease of using the system was not a concern for the participants. During the study process, the participants rarely reported problems related to the user interface. This suggests that placing excessive emphasis on interface appearance or aesthetics and operational convenience is unnecessary when considering the design of the PHR system; instead, the design should emphasize other factors patients truly value, such as the appropriateness of the system functions. 

### 5.4. Effect of PU on BI

In the field of healthcare, many TAM studies have verified that an individual’s willingness to use an information system is determined by the system’s usefulness to them [67]. This result is supported by Jian’s investigation [10] on consumers’ adoption of USB-based PHR and with previous other studies of patients’ acceptance of technology [36,58]. PU was found herein to be the most significant factor, and to explain a significant fraction of the variance in behavioral intentions. However, Winkelman and colleagues [22] claimed that, for patients with chronic inflammatory bowel disease (IBD), simply providing access to EMRs was of little use. They determined that useful information technology for patients with IBD should provide the following four things; a sense of ownership of the illness, patient-driven communication, personalized support, and mutual trust. On the other hand, according to the feedbacks from non-responded participants indicated that the major reason, which they cannot complete the study is they are too busy and forget to use. This may also implicate that they felt that the PHR system is useless, resulting their unwillingness to use.

The PHR system that was developed in this study has an important role in helping patients maintain routine self-completed health records that can be checked by medical professionals, who can then interact with their patients, resulting in, hopefully, timely and appropriate interventions. Thus, from the perspective of Winkelman and colleagues [22], the PHR is a useful technology. The results herein suggest that PHR design should primarily seek to develop functions that satisfy the patients’ requirements with the intention of motivating them to use the system. 

### 5.5. Effect of PPR on BI

In this study, PPR was significantly related to BI, as expected. This result is consistent with the results of some empirical studies of healthcare environments [44,45]. When physicians and patients maintain a better relationship, the patients have a stronger intention to consult with, and provide complete information to physicians to facilitate further positive interaction and to obtain a high-quality diagnosis. The PHR that is developed in this study can be regarded as a “physician-driven” system for patients if use of the PHR is part of the healthcare plan that is devised by the physician for the patients with a view to maintaining an interactive relationship, then patients will be more willing to use it. Therefore, PPR can be expected to influence PHR use, suggesting that a higher PPR is associated with greater intention to follow the physician’s instructions to use the PHR.

During the study period, the system accumulated hundreds of records, including the patient’s health records and the health staffs’ responses to patients. This shows that both physicians and patients has jointly experienced the PHR system, no matter whether patients provided their health records to the care staffs or read care staffs’ interpretation about each of their relaxation practice. This may also echo the physician-patient relationship plays a critical role to affect patients’ intention for using the PHR system. This suggests that the higher the PPR a patient perceives, the higher their intention to follow the physician’s suggestions (or orders) to use the PHR will be.

From the perspective of healthcare institutions, the purpose of developing a PHR system is to improve the quality of healthcare using technology. However, if the PPR is poor, then patients will tend to have a low intention to interact with medical professionals using the system, defeating the purpose of its development. Based on the findings herein, we suggest that healthcare institutions should select patients and physicians that have superior PPRs to promote the use of the PHR systems and to encourage medical teams to enhance PPRs whenever possible to promote the use of the PHR systems. 

## 6. Conclusions

### 6.1. Summary of Findings and Contributions

In this study, the PHR system was implemented as a telehealthcare tool to be an auxiliary therapeutic tool by infertility and mental health professionals. The results of this study provide initial insights into the functions that should be provided by a PHR system for infertility and, specifically, on the determinants of PHR acceptance by patients. Patients’ acceptance of the PHR was examined using the TAM. The application of the seldom-discussed concept, PPR, enhanced the power of the TAM to predict patients’ behavioral intentions to use PHR. As in several TAM studies of healthcare, the results of this study indicated that PEOU did not significantly affect patients’ behavioral intentions. Therefore, this study contributes to the growing number of TAM studies on special-purpose systems, such as healthcare systems, and on the acceptance of technology, by demonstrating that PEOU is not a significant determinant of BI. Moreover, PPR was introduced as a rarely discussed but meaningful determinant of the BI of the patients. 

The delivery of healthcare is being transformed by advances in e-health and by an empowered, computer-literate public [68]. However, interactive information systems, such as the PHR, developed for both patients and physicians, remain in their early stages of development [69]. Based on the findings herein, we advocate ongoing exploration of patients’ acceptance of such technology to enhance healthcare outcomes.

### 6.2. Limitations and Future Directions

Although a rigorous research model design, instrument development, sample recruitment, and data analysis were utilized herein, this study has several limitations. First, a single physician treated all participating patients, comprising a small sample, at one hospital, possibly affecting the representativeness and generalizability of the results. Second, the responses to the self-report questionnaires may have been affected by each participant’s perception of the questionnaire items (*i.e.*, common method bias).

In this study, PPR was validated to be a motivator of patients’ use of healthcare information systems; however, a cyclic effect may exist between physician-patient relationship and behavioral intention to use healthcare information systems [68]. Various studies have found that, in the long run, the use of healthcare information technology can improve the interaction between physicians and patients, increasing (*i.e.*, PPR) [70,71,72]. Further research is required to explore how the PU, PEOU, and other factors influence PPR. Additionally, the TAM suggests that users’ BI towards a technology affects their actual use of it; therefore, finding appropriate indicators of the actual objective use of the PHR is a worthy goal of research based on longitudinal observations. Based on this study, the degree of reduction of depression and improvement of care quality are possible indicators. Together, PU, PEOU, and PPR accounted for 57.7% of the variance of BI to use PHR, implying that other factors, such as *trust* perception [73], which can promote the use) of TAM in the field of healthcare, should be sought. Moreover, recruiting various types of patients is recommended to increase the representativeness and generalizability of the results.
